# Reaction rate of pyruvate and hydrogen peroxide: assessing antioxidant capacity of pyruvate under biological conditions

**DOI:** 10.1038/s41598-019-55951-9

**Published:** 2019-12-20

**Authors:** Victoria A. Guarino, William M. Oldham, Joseph Loscalzo, Ying-Yi Zhang

**Affiliations:** Department of Medicine, Brigham and Women’s Hospital, Harvard Medical School, Boston, MA 02115 USA

**Keywords:** Cardiovascular biology, Chemical biology, Pharmacology

## Abstract

Pyruvate, a pivotal glucose metabolite, is an α-ketoacid that reacts with hydrogen peroxide (H_2_O_2_). Its pharmacological precursor, ethyl pyruvate, has shown anti-inflammatory/anti-tissue injury effects in various animal models of disease, but failed in a multicenter clinical trial. Since rodents, but not humans, can convert ethyl pyruvate to pyruvate in blood plasma, this additional source of extracellular pyruvate may have contributed to the discrepancy between the species. To examine this possibility, we investigated the kinetics of the reaction under biological conditions and determined the second order rate constant *k* as 2.360 ± 0.198 M^−1^ s^−1^. We then calculated the time required for H_2_O_2_ elimination by pyruvate. The results show that, with an average intracellular concentration of pyruvate (150 µM), elimination of 95% H_2_O_2_ at normal to pathological concentrations (0.01–50 µM) requires 141–185 min (2.4–3 hour). With 1,000 µM pyruvate, a concentration that can only exist extracellularly or in cell culture media, 95% elimination of H_2_O_2_ at 5–200 µM requires 21–25 min. We conclude that intracellular pyruvate, or other α-ketoacids, whose endogenous concentration is controlled by metabolism, have little role in H_2_O_2_ clearance. An increased extracellular concentration of pyruvate, however, does have remarkable peroxide scavenging effects, considering minimal peroxidase activity in this space.

## Introduction

In recent years, ethyl pyruvate, an ethyl ester of pyruvate, has been shown to exert protective effects in various rodent models of acute/chronic inflammation and oxidant-induced tissue/organ injury^1^. These effects, however, were not observed in humans as examined in a phase II clinical trial^2^. This discrepancy between animals and humans might be related to the different *in vivo* activation site of ethyl pyruvate, and its interaction with hydrogen peroxide (H_2_O_2_) in each environment. This study investigated the reaction rate of pyruvate and H_2_O_2_ under these conditions to evaluate such differences.

**Figure 1:**
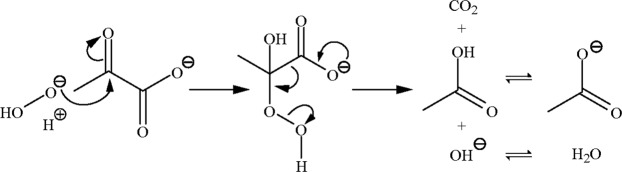
Mechanism of pyruvate and H_2_O_2_ reaction. Nucleophilic addition of H_2_O_2_ to the α-carbonyl group in pyruvate forms an unstable intermediate, 2-hydroperoxy-2-hydroxypropanoate, which subsequently undergoes rearrangement to produce CO_2_, acetate, and water at neutral pH.

The reaction of pyruvate and H_2_O_2_ produces acetate, carbon dioxide (CO_2_), and water; its transition intermediate has been recently confirmed^3,4^. As shown in Fig. 1, the intermediate is an adduct of H_2_O_2_ and pyruvate, and its subsequent rearrangement is initiated by the release of CO_2_ from the carboxyl group. In ethyl pyruvate, ester formation blocks the pyruvate carboxyl and, therefore, the rearrangement cannot proceed to form these products. Ethyl pyruvate can be activated *in vivo* by carboxylesterase, an esterase with specificity towards carboxylate. Carboxylesterase is widely distributed within mammalian cells. In blood plasma, however, it is abundant in rodents but absent in humans^5–7^. Thus, ethyl pyruvate activation occurs in the plasma of rodents once absorbed or injected, but only in the intracellular environment of human cells.

Hydrogen peroxide is formed by superoxide dismutation, with superoxide arising from two primary sources: electron leak along the electron transport chain in mitochondria and the enzymatic action of NADPH oxidases (NOXs)^8^. NOXs are mainly localized to the plasma membrane and release superoxide/H_2_O_2_ at high concentrations to the extracellular space. Hydrogen peroxide enters cells through aquaporin transporters, and is removed by three peroxidase systems: glutathione peroxidases, catalase, and the peroxiredoxins. The average intracellular concentration of H_2_O_2_ is less than 10 nM, but that of blood is 1–5 µM^9^. Under inflammatory conditions, plasma H_2_O_2_ concentrations can reach 50 µM^9^.

The concentration of pyruvate is 77–201 µM intracellularly^10–12^, and 47–118 µM in plasma^11,13,14^. As a pivotal metabolite, pyruvate is continuously produced and removed by various metabolic pathways: after being produced by glycolysis, pyruvate is decarboxylated to acetyl CoA, which can either enter the tricarboxylic acid (TCA) cycle and generate ATP via oxidation, or participate in fatty acid synthesis. Pyruvate can also be carboxylated to oxaloacetate, which replenishes the TCA cycle or enters the gluconeogenesis pathway to generate glucose. Transamination converts pyruvate to alanine, which participates in protein synthesis. Under oxygen-deficient conditions, pyruvate is reduced to lactate. These pathways comprise regulated enzymatic reactions, which result in maintaining a relatively constant concentration of pyruvate intracellularly.

In the present study, we first examined the reaction order of pyruvate and H_2_O_2_ to confirm the concentration dependence of the reactants. The rate constant (*k*) was then determined, since there are no reports in the literature of the *k* obtained under physiological temperature, pH, and ionic strength. With this biomarker rate constant in hand and the biologically relevant concentrations of H_2_O_2_ and pyruvate described above, the rates of H_2_O_2_ elimination by pyruvate were calculated and confirmed by experimental measurements.

## Results

### Reaction order with respect to pyruvate and H_2_O_2_

The rate law of the pyruvate and H_2_O_2_ reaction is shown in Eq. (1), in which [Pyr] and [H_2_O_2_] represent the concentrations of pyruvate and H_2_O_2_, respectively; *k* is the rate constant; and a and b are the reaction orders with respect to pyruvate and H_2_O_2_, respectively.

1 $$\frac{-d[Pyr]}{dt}{\rm{or}}\frac{-d[{{\rm{H}}}_{2}{{\rm{O}}}_{2}]}{dt}=k\,{[{\rm{Pyr}}]}^{{\rm{a}}}{{[{\rm{H}}}_{2}{{\rm{O}}}_{2}]}^{{\rm{b}}}$$

**Figure 2:**
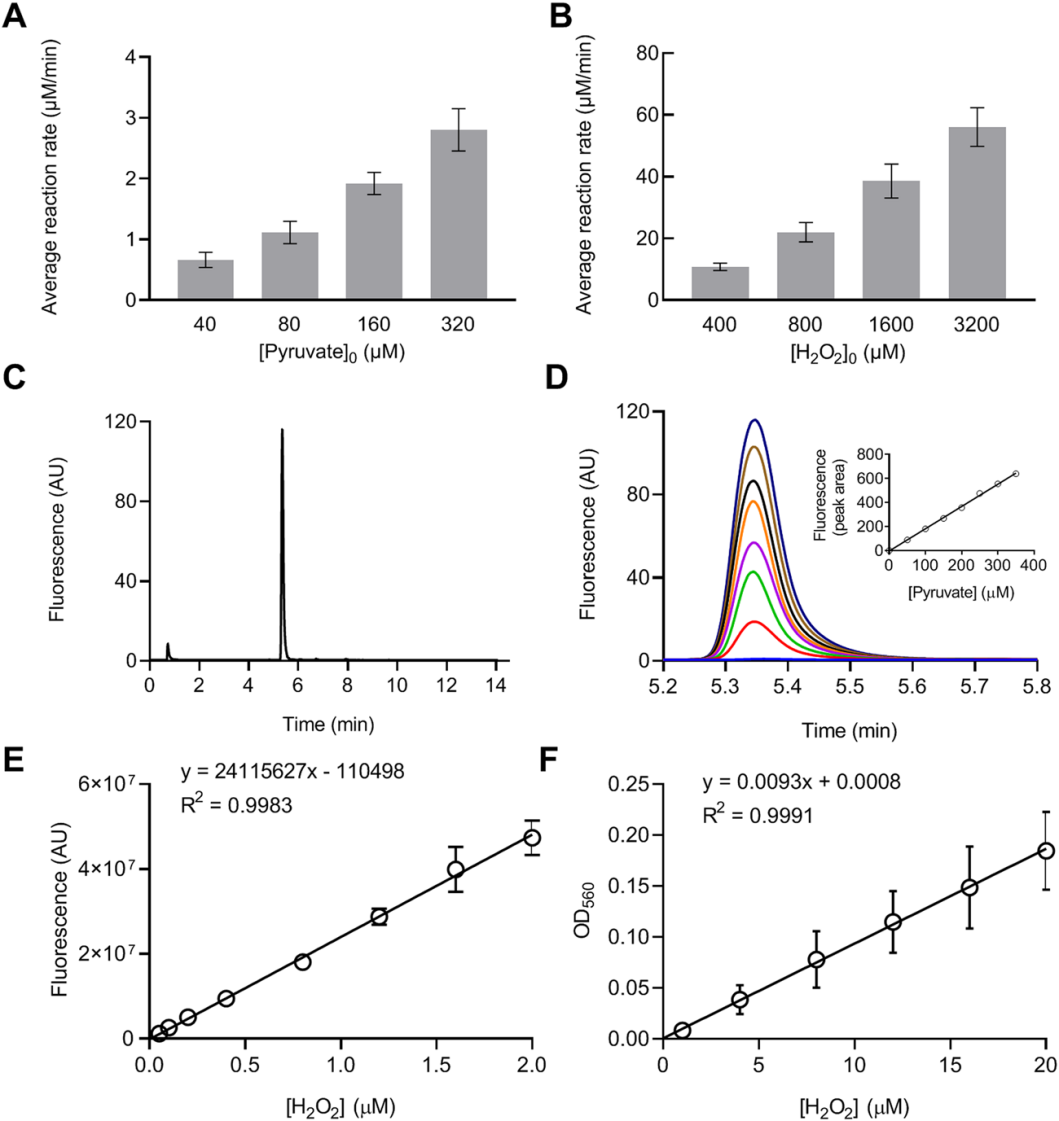
Reaction order with respect to pyruvate and H_2_O_2_. **(A**) The reaction order with respect to pyruvate was analyzed by reacting 20 µM H_2_O_2_ with increasing concentrations of pyruvate. The reaction was carried out for 5 min and the average reaction rate was calculated as the change of H_2_O_2_ concentration per min. **(B)** The reaction order of H_2_O_2_ was determined by reacting 200 µM pyruvate with increasing concentrations of H_2_O_2_. The reaction was carried out for 3 min and the rate was calculated as the change in pyruvate concentration per min. **(C)** and **(D)** HPLC chromatograms and the standard curve for pyruvate. **(E)** and **(F)** Standard curves of H_2_O_2_ measurement by a peroxidase-Amplex Red assay. The color product of the assay was read by absorption at OD_560_ or by fluorescence at λ_ex_ 350 nm and λ_em_ 410 nm for the high or low concentration range of H_2_O_2_, respectively. Data were obtained from 3 separate experiments and presented as mean ± SD.

To evaluate the reaction under biological conditions, experiments in this report were all carried out at 37 °C in Dulbecco’s phosphate-buffered saline (DPBS), which has a pH of 7.3 ± 0.2 and ionic strength of 165 mM. The reaction order of pyruvate was estimated by reacting a fixed concentration of H_2_O_2_ (20 µM) with increasing concentrations of pyruvate (40, 80, 160, and 320 µM) for 5 min. Hydrogen peroxide concentration in the solutions was subsequently measured by a peroxidase-Amplex Red colorimetric assay. As shown in Fig. 2A, the increase in the average reaction rate was approximately proportional to the increase in pyruvate concentration, indicating that the reaction was first order with respect to pyruvate, or a =1. To determine the reaction order of H_2_O_2_, 200 µM pyruvate was reacted with increasing concentrations of H_2_O_2_ (400, 800, 1600, or 3200 µM) for 3 min. Pyruvate concentration was then measured using an HPLC method. The average reaction rate was found to increase proportionally to that of H_2_O_2_ concentration, indicating a first order reaction with respect to H_2_O_2_, or b = 1 (Fig. 2B). Taken together, the overall reaction order is two. Note that the reaction rate was measured as an average rate rather than an instantaneous rate and is less accurate than the latter; this approach should not, however, affect the estimation of the reaction order. As a demonstration of the method used for the above measurements, Fig. 2C,D show the HPLC chromatograms of the pyruvate and its standard curve. Pyruvate was derivatized to become fluorescent prior to HPLC analysis. Hydrogen peroxide was measured by a peroxidase-Amplex Red assay. The color product of the assay exhibits both fluorescence and visible color. Hydrogen peroxide samples at low concentration range (0–2 µM) were read with fluorescence and high concentrations (0–20 µM) with absorbance (OD_560_) in this assay. The standard curves in both ranges are linear (Fig. 2E,F).

### The rate constant of pyruvate and H_2_O_2_ reaction

To determine the rate constant *k* of the reaction, Eq. (1) was transformed to Eq. (2), in which [Pyr] represents the concentration of both reactants, and [Pyr] = [H_2_O_2_]. Integration of Eq. (2) from [Pyr]_0_ to [Pyr] and from 0 to *t* gives Eq. (3):

2 $$\frac{-d[Pyr]}{dt}=k\,{[{\rm{Pyr}}]}^{2}$$

3 $$\frac{1}{[{\rm{Pyr}}]}={kt}+\frac{1}{{[{\rm{Pyr}}]}_{0}}$$

**Figure 3:**
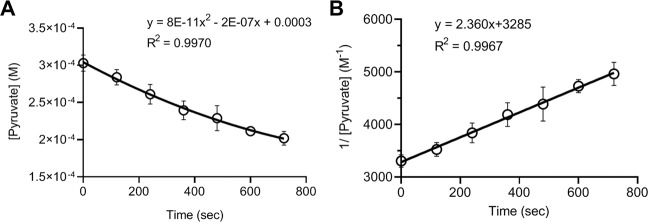
Rate constant of pyruvate and H_2_O_2_ reaction. Reactions were carried out with 300 µM each of pyruvate and H_2_O_2_ in DPBS at 37 °C. At the indicated timepoint, pyruvate concentration in the reaction solution was measured using the HPLC method. **(A)** Data are graphed as pyruvate concentration vs. time and fit with a second-degree polynomial equation **(B)** Data are graphed as inverse pyruvate concentration vs. time and fit with a linear equation. The slope of the linear line is the rate constant *k* of the reaction according to Eq. (3). Data were obtained from 6 separate experiments and presented as mean ± SD.

The rate constant *k* was determined by reacting pyruvate with H_2_O_2_, at the initial concentration of 300 µM, for 0, 2, 4, 6, 8, 10, and 12 min; pyruvate concentration was then measured by the HPLC analysis. As shown in Fig. 3, pyruvate concentration decreased over time, which fits a second-degree polynomial equation (Fig. 3A); plotting the inverse concentration of pyruvate vs. time gives a linear relationship, with a slope of 2.360 and an intercept of 3285 (Fig. 3B). Compared to Eq. (3), the plot indicates that the reaction rate constant *k* is 2.360 M^−1^ s^−1^, with [Pyr]_0_ is 0.000304 M or 304 µM by this measurement. Figure 3 was plotted with data sets from 6 replicate experiments. When graphed individually, the average and standard deviation of the *k* was 2.360 ± 0.198 M^−1^ s^−1^, or 0.000142 ± 0.000012 µM^−1^ m^−1^.

**Figure 4:**
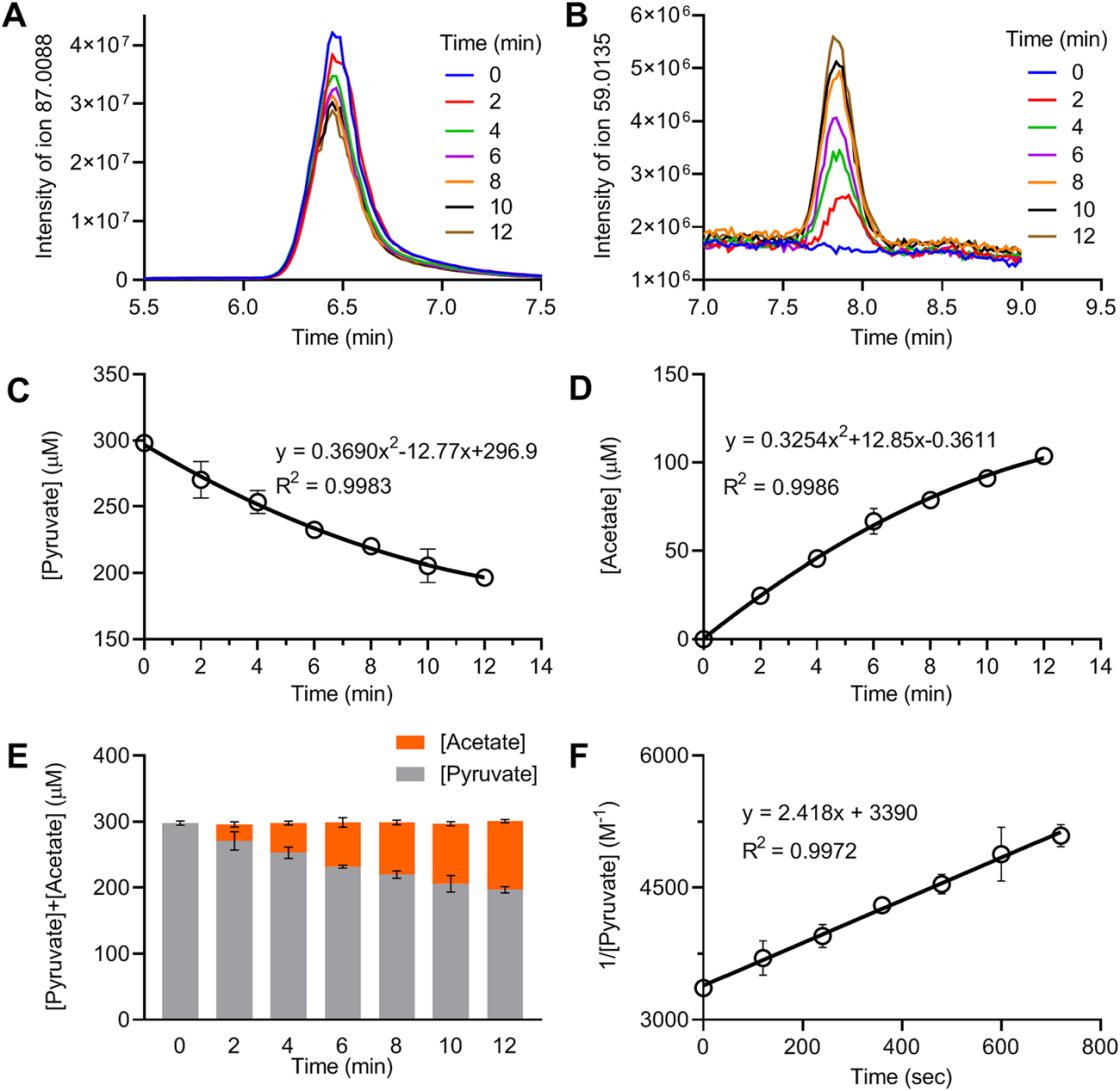
LC-MS measurement of pyruvate and acetate concentrations. Reactions were carried out with 300 µM each of pyruvate and H_2_O_2_ in DPBS at 37 °C. At the indicated time, aliquots of the reaction mixture were removed for LC-MS analysis. **(A**,**B)** LC-MS chromatograms showing the detection of pyruvate and acetate, respectively, in the samples at the indicated times. **(C**,**D)** Concentrations of pyruvate and acetate, respectively, were calculated according to standard curves, graphed vs. time, and fit to second-degree polynomial equations. **(E)** Data are graphed as the sum of pyruvate and acetate concentrations vs. time, showing an unchanged total concentration over time. **(F)** Data are graphed as the inverse concentration of pyruvate vs. time and fit to a linear equation. The slope of the line indicates the *k* of the reaction. Data were obtained from 3 separate experiments and presented as mean ± SD.

To confirm these HPLC measurements, a LC-MS method was used to measure the concentrations of pyruvate and acetate in the reaction solutions. As shown in Fig. 4A,B, the LC-MS chromatograms demonstrated distinct peaks for both pyruvate and acetate in the samples obtained at various timepoints. The decrease in pyruvate concentration over time was associated with an increase in the production of acetate (Fig. 4C,D). The total concentrations of pyruvate and acetate at each time point were constant (Fig. 4E), indicating an equimolar conversion of pyruvate to acetate. Plotting the inverse concentration of pyruvate over time showed a linear relationship with a slope of 2.418 and intercept of 3390 (Fig. 4F), consistent with the *k* as 2.418 M^−1^ s^−1^ and [Pyr]_0_ as 295 µM by the LC-MS measurement.

### Rate of H_2_O_2_ elimination by pyruvate under biological conditions

As mentioned above, the *in vivo* concentration of pyruvate is greater than that of H_2_O_2_. To calculate the rate of H_2_O_2_ elimination, Eq. (1) was transformed into Eq. (4), in which *x* represents the amount of the reactants that had undergone transformation.

4 $$\frac{dx}{dt}={k}({[{\rm{Pyr}}]}_{0}-x{)([{\rm{H}}}_{2}{{\rm{O}}}_{2}{]}_{0}-x)$$

The integration of Eq. (4) from 0 to *t* and from 0 to *x* gives Eq. (5), which allows for the calculation of the time required for *x* amount of H_2_O_2_ to be reacted.

5 $$t=\frac{1}{{{[{\rm{H}}}_{2}{{\rm{O}}}_{2}]}_{0}-{[{\rm{Pyr}}]}_{0}}(\mathrm{ln}\,\frac{({{[{\rm{H}}}_{2}{{\rm{O}}}_{2}]}_{0}-x){[{\rm{Pyr}}]}_{0}}{({[{\rm{Pyr}}]}_{0}-x){{[{\rm{H}}}_{2}{{\rm{O}}}_{2}]}_{0}})\frac{1}{k}$$

Rearranging Eq. (5) gives Eq. (6), which allows for the calculation of the amount of H_2_O_2_ reacted at a specified time.

6 $$x=\frac{{[{\rm{Pyr}}]}_{0}{{[{\rm{H}}}_{2}{{\rm{O}}}_{2}]}_{0}-{[{\rm{Pyr}}]}_{0}{{[{\rm{H}}}_{2}{{\rm{O}}}_{2}]}_{0}{e}^{kt({{[{\rm{H}}}_{2}{{\rm{O}}}_{2}]}_{0}-{[{\rm{Pyr}}]}_{0})}}{{[{\rm{Pyr}}]}_{0}-{{[{\rm{H}}}_{2}{{\rm{O}}}_{2}]}_{0}{e}^{kt({{[{\rm{H}}}_{2}{{\rm{O}}}_{2}]}_{0}-{[{\rm{Pyr}}]}_{0})}}$$

For calculations of the rate of H_2_O_2_ elimination with various amounts of [Pyr]_0_ and [H_2_O_2_]_0_, Excel spreadsheets were used with the above equations embedded. Examples are shown in Tables 1 and 2.

**Table 1:** Calculation of time required for H_2_O_2_ elimination at indicated percentage*.

*k*	[Pyr]_0_	[H_2_O_2_]_0_	R	*x*	[Pyr]	[H_2_O_2_]	P	Q	*t*
(µM^−1^ m^−1^)	(µM)	(µM)	(%)	(µM)	(µM)	(µM)			(min)
0.000142	150	50	50	25	125	25	−0.010	−0.511	36.08
0.000142	150	50	95	47.5	103	2.5	−0.010	−2.615	184.67
0.000142	1,000	50	50	25	975	25	−0.001	−0.668	4.96
0.000142	1,000	50	95	47.5	953	2.5	−0.001	−2.947	21.91

*The values for *k*, [Pyr]_0_, [H_2_O_2_]_0_, and R were entered, R indicates the percentage of H_2_O_2_ elimination. The remaining terms are calculated using the following equations: *x* =  R*[H_2_O_2_]_0_; [Pyr] = [Pyr]_0_ *−* *x*; [H_2_O_2_] = [H_2_O_2_]_0_ *−* *x*; P = $\frac{1}{{{[{\rm{H}}}_{2}{{\rm{O}}}_{2}]}_{0}-{[{\rm{Pyr}}]}_{0}}$; Q = $\mathrm{ln}\,\frac{({{[{\rm{H}}}_{2}{{\rm{O}}}_{2}]}_{0}-x){[{\rm{Pyr}}]}_{0}}{({[{\rm{Pyr}}]}_{0}-x){{[{\rm{H}}}_{2}{{\rm{O}}}_{2}]}_{0}}$; and *t* = P*Q/*k*.

**Table 2:** Calculation of the amount of H_2_O_2_ eliminated (x) at indicated time t.

*k*	*t*	[Pyr]_0_	[H_2_O_2_]_0_	D	E	*x*	[H_2_O_2_]
(µM^−1^ m^−1^)	(min)	(µM)	(µM)	(µM)		(µM)	(µM)
0.000142	33.0	150	50	−100	0.6267	23.59	26.41
0.000142	22.0	1,000	50	−950	0.0518	47.53	2.47

*The values for *k*, *t*, [Pyr]_0_, and [H_2_O_2_]_0_ were entered. The remaining terms are calculated using the following equations: D = [H_2_O_2_]_0_ − [Pyr]_0_; E = ${e}^{ktD}$; $x=\frac{({[{\rm{Pyr}}]}_{0}{{[{\rm{H}}}_{2}{{\rm{O}}}_{2}]}_{0}-{[{\rm{Pyr}}]}_{0}{{[{\rm{H}}}_{2}{{\rm{O}}}_{2}]}_{0}{\rm{E}}}{{[{\rm{Pyr}}]}_{0}-{{[{\rm{H}}}_{2}{{\rm{O}}}_{2}]}_{0}{\rm{E}}}$; and [H_2_O_2_] = [H_2_O_2_]_0_ − *x*.

**Figure 5:**
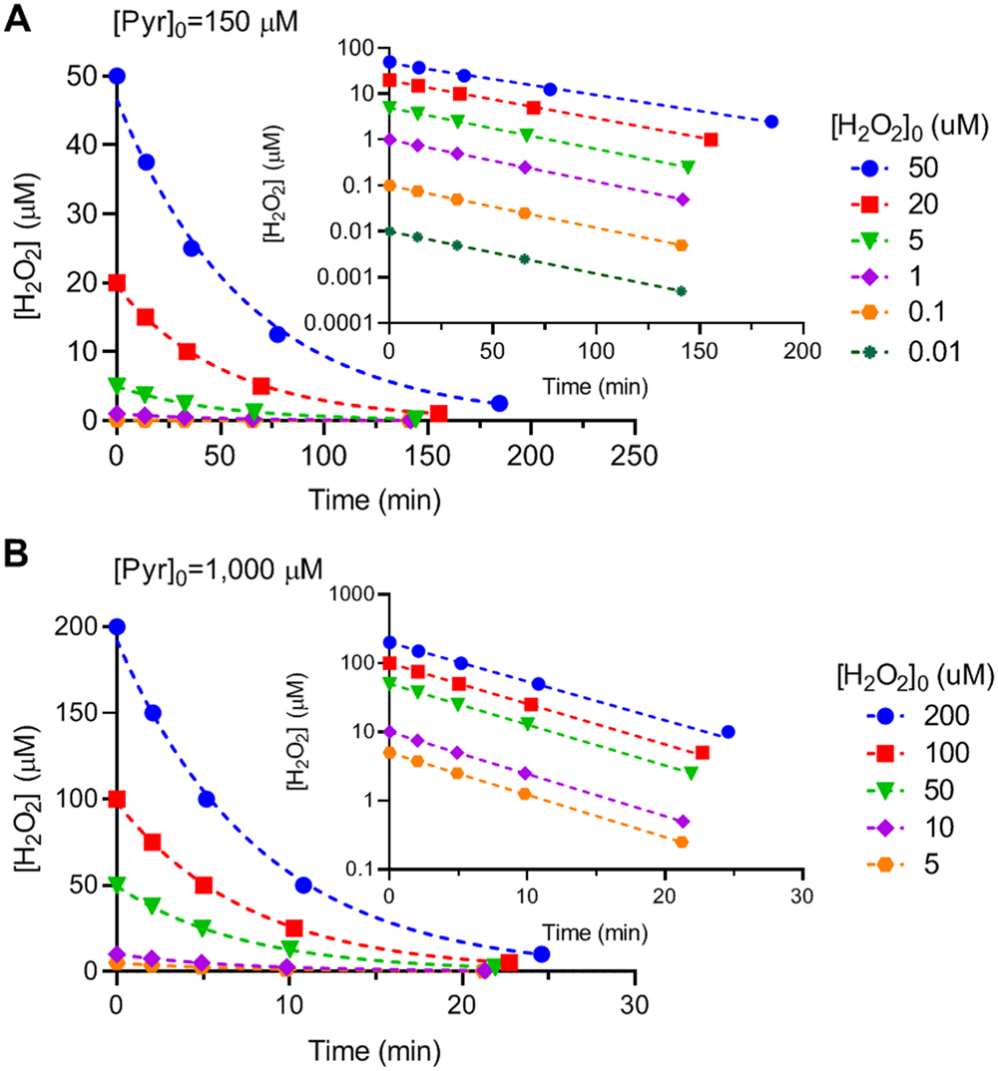
Calculated time course of H_2_O_2_ elimination by pyruvate. The time required for elimination of 25, 50, 75, or 95% of H_2_O_2_ of its initial concentration by pyruvate was calculated based on Eq. (5) and Table 1. **(A)** The initial concentration of pyruvate is 150 µM and of [H_2_O_2_]_0_ is 50, 20, 5, 1, 0.1, or 0.01 µM. **(B)** The initial concentration of pyruvate is 1,000 µM and of [H_2_O_2_]_0_ is 200, 100, 50, 10, or  5 µM. Data are graphed as [H_2_O_2_] vs. time, and fit to exponential decay equations. Inserts show the same graphs on a semilog scale.

The time for elimination of 25, 50, 75, and 95% of H_2_O_2_ of its initial concentration, [H_2_O_2_]_0_, was calculated with Eq. (5). Two initial pyruvate concentrations were used: a normal intracellular concentration, 150 µM, and an increased extracellular concentration, 1,000 µM. Each concentration of pyruvate was reacted with serial concentrations of H_2_O_2_, ranging from normal to extreme pathological concentrations. The calculated results are shown in Fig. 5. The data were fitted to exponential decay equations, as [Pyr]_0_ was much greater than [H_2_O_2_]_0_ in each set of reactions. At the concentration differences of ≥10 fold (the lower four values of [H_2_O_2_]_0_ in each set), the reactions became pseudo-first order and the R^2^ of the data fitting ≥0.9998. As shown in Fig. 5A, at [Pyr]_0_ = 150 µM, the time required for eliminating 25, 50, 75, and 95% of H_2_O_2_ with initial concentrations of 0.01–50 µM ranged as 13.5–14.2, 33–36, 65–78, and 141–185 min, respectively. At [Pyr]_0_ = 1,000 µM (Fig. 5B), the time required for eliminating 25, 50, 75, and 95% H_2_O_2_ at initial concentrations of 5–200 µM ranged as 2.0–2.1, 4.9–5.2, 10–11, and 21–25 min, respectively.

To confirm these calculations, experiments were carried out by reacting 150 µM pyruvate with 0.1, 1, 5, or 50 µM H_2_O_2_. After a 33 min incubation, the H_2_O_2_ concentration in the reaction solution was measured. The H_2_O_2_ concentrations were also calculated under these conditions using the method shown in Table 2. The measured values were consistent with what was calculated (Table 3). Similarly, 1,000 µM pyruvate was reacted with 5, 10, 50, 100, and 200 µM H_2_O_2_ for 22 min, and the H_2_O_2_ concentrations were subsequently measured and compared with calculated values (Table 3).

**Table 3:** Comparison of measured and calculated H_2_O_2_ concentrations at indicated time of reaction*.

[Pyr]_0_	[H_2_O_2_]_0_	Reaction time	Measured [H_2_O_2_]	Calculated [H_2_O_2_]
(µM)	(µM)	(min)	(µM)	(µM)
150	0.1	33	0.05 ± 0.02	0.05
150	1	33	0.50 ± 0.06	0.50
150	5	33	2.74 ± 0.24	2.50
150	50	33	27.2 ± 1.64	26.4
1,000	5	22	0.22 ± 0.07	0.22
1,000	10	22	0.39 ± 0.08	0.45
1,000	50	22	3.06 ± 0.33	2.47
1,000	100	22	5.95 ± 0.35	5.49
1,000	200	22	16.2 ± 1.11	13.5

*Measured data were obtained from 3 separate experiments and are presented as mean ± SD.

**Figure 6:**
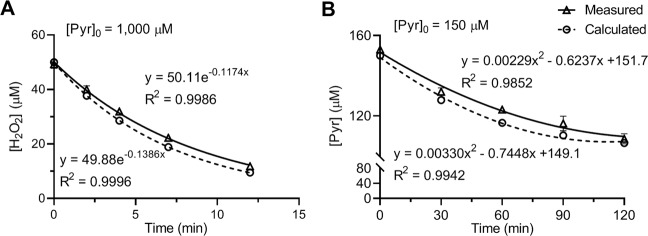
Comparison of measured and calculated time course of pyruvate and H_2_O_2_ reaction. Reactions were carried out by reacting 1,000 µM **(A)** or 150 µM **(B)** pyruvate with 50 µM H_2_O_2_ in DPBS at 37 °C. Concentrations of H_2_O_2_ **(A)** or pyruvate **(B)** in the reaction solutions were measured at indicated timepoints. Calculated [H_2_O_2_] or [Pyr] values were obtained based on Eq. 6 and Table 2. Data were fitted to exponential decay **(A)** and to second-degree polynomial **(B)** equations as shown within the plots. Measured data were obtained from 3 separate experiments and are presented as mean ± SD.

Confirmation was also carried out by reacting 50 µM H_2_O_2_ with 1,000 µM (Fig. 6A) or 150 µM pyruvate (Fig. 6B) and measuring the concentrations of H_2_O_2_ and pyruvate, respectively, after various incubation times. The corresponding time course was calculated according to Table 2. As shown in the figure, the measured and calculated time courses of the reaction aligned with each other. The data sets were fitted to exponential decay equations in Fig. 6A and second-degree polynomial equations in Fig. 6B.

## Discussion

This study examined the reaction rate of pyruvate and H_2_O_2_ under biological conditions. The reaction was determined to be first-order with respect to each reactant (Fig. 2), and the second order reaction rate constant *k* was 2.360 ± 0.198 M^−1^ s^−1^ or 0.000142 ± 0.000012 µM^−1^ m^−1^ (Figs. 3 and 4). Reaction rates were calculated with the determined *k* value using the concentrations of pyruvate and H_2_O_2_ observed under various biological conditions. The analysis showed that in the presence of an average intracellular concentration of pyruvate (150 µM), 50% elimination of H_2_O_2_ at normal to pathological concentrations (0.01–50 µM) requires 33–36 min, and 95% elimination takes 141–185 min (2.4–3 hr). In the presence of 1,000 µM pyruvate, 50% elimination of H_2_O_2_ at 5–200 µM requires only 5 min (4.9–5.2 min), and 95% elimination takes 21–25 min (Fig. 5). Experimental measurements confirmed these calculations (Table 3 and Fig. 6).

These data indicate that intracellular pyruvate has little role in H_2_O_2_ clearance. The rate of H_2_O_2_ elimination by 150 µM pyruvate (95%, 2.4–3 h) is too slow compared with intracellular peroxidase activities: The second order rate constants for reactions between H_2_O_2_ and glutathione peroxidases, catalase, or peroxiredoxins are in the 10^7^–10^8^ M^−1^ s^−1^ range^15–17^. Taking cytosolic glutathione peroxidase (GPx-1) alone, at a concentration of ~2 µM^18^ and rate constant of 4.1 ×10^7^ M^−1^ s^−1^ ^17^, the enzyme eliminates 95% of 1 µM H_2_O_2_ in 0.06 seconds, as calculated with Eq. 5 or Table 1. The same extent of elimination by 150 µM pyruvate requires 8,501 seconds (2.36 h). Low-molecular-weight thiols can also react with H_2_O_2_ directly, but the reaction rates are similar to that of pyruvate (within an order of magnitude). The second order rate constants for glutathione and cysteine, for example, are 0.89 and 2.9 M^−1^ s^−1^ (pH 7.4, 37 °C), respectively^19^. At average intracellular concentrations of 1–2 mM^20^ and 20–100 µM^21^, glutathione and cysteine eliminate 95% of 1 µM H_2_O_2_ in 1,684–3,368 seconds (0.5–0.9 h) and 10,401–53,486 seconds (2.9–14.9 h), respectively, as calculated with Eq. 5 or Table 1.

In the extracellular space, the rate of H_2_O_2_ elimination by 1,000 µM pyruvate (95%, 21–25 min) is rapid, considering minimal peroxidase activities present in this compartment and a 100- to 500-fold higher H_2_O_2_ concentration in plasma compared to the intracellular environment. Inflammation-activated NOXs can result in an extracellular accumulation of H_2_O_2_. Removing this pool of extracellular H_2_O_2_ by pyruvate would attenuate the oxidative stress/injury on the cells.

Ethyl pyruvate has been administered to mice and rats at doses of 40–100 mg/kg^1^, which would translate to blood concentrations of 4.4–12.3 mM using a blood-to-body weight ratio of 7%^22^. If ethyl pyruvate is activated by carboxylesterase in blood plasma, it will achieve an extracellular concentration close to 1,000 µM. At this concentration, pyruvate can markedly reduce the extracellular H_2_O_2_ level before entering cells to be metabolized. By contrast, when ethyl pyruvate can only be activated intracellularly (due to lack of plasma carboxylesterase), the generated pyruvate would be rapidly metabolized, having little impact on H_2_O_2_ levels. Thus, the conflicting results of the effects of ethyl pyruvate comparing rodent studies and the human trial may be related to the site of *in vivo* activation of the compound.

Based on the mechanism shown in Fig. 1, pyruvate could react with lipid peroxides and peroxynitrite in the same manner, forming an α-carbon adduct intermediate and releasing lipid hydroxide and nitrite as products, respectively. Thus, pharmacological concentrations of pyruvate could also scavenge these reactive oxygen species in the cell membrane, as well as other compartments. The same mechanism could also apply to other α-ketoacids, e.g., α-ketoglutarate and oxaloacetate. Nevertheless, the rate constant for these α-ketoacids would be lower than that of pyruvate owing to a bulkier alkyl chain^23^. The low intracellular concentrations of these metabolites, as with pyruvate, would, therefore, prevent them from having any practical “antioxidant” effects in that compartment.

It is also worthwhile noting that pyruvate is vital for primary cell culture and is added to various cell culture media at a concentration of 1,000 µM. Many oxidative stress studies add H_2_O_2_ to culture medium as an oxidant stress for cells, usually with an extended incubation period. As the added H_2_O_2_ is eliminated rapidly by pyruvate in these media, 50% in 5 min and 95% in 25 min, caution must be taken in the interpretation of the results, especially when comparing different studies.

## Methods

### Materials

Sodium pyruvate (≥99% purity, #P2256), 30% hydrogen peroxide (#H1009), acetic acid (#338826), sodium acetate trihydrate (#71188-250 G), O-phenylenediamine (#P23938), catalase (#C40), and Dulbecco’s phosphate buffered saline (DPBS, #D8537) were purchased from Sigma. Formic acid (#A11750), 3% hydrogen peroxide (#AC426001000), Amplex® Red hydrogen peroxide/peroxidase assay kit (#A22188), HPLC grade water (#W5-4), and HPLC grade acetonitrile (#A955-4) were obtained from Thermo Fisher Scientific.

### Reaction of pyruvate and H_2_O_2_

Pyruvate and H_2_O_2_ reactions were carried out in DPBS at 37 °C in a total volume of 5 mL. Before the reaction, DPBS was incubated in a 37 °C water bath for at least 30 min to attain a stable temperature. Small aliquots of pyruvate and H_2_O_2_ stock solutions (<0.2 mL) were added to the DPBS solution to achieve the final concentration. Reactions were carried out at 37 °C for the designated times, and aliquots were removed for subsequent analysis. Owing to the time-sensitivity of the measurements, mixing and removing the reaction solution was staggered in order. For the measurements of pyruvate and acetate concentrations (by HPLC or LC-MS methods, see below), the reactions were stopped by adding 20 units catalase (10 unit/µL) to a 150 µl reaction mixture. For measurement of H_2_O_2_ concentration (by peroxidase-Amplex Red method, see below), aliquots of the reaction solution were directly loaded into a 96 well plate and immediately mixed with horseradish peroxidase and Amplex Red reagents to stop the reaction. Corresponding standards were included in each experiment and processed in the same way as samples.

### HPLC measurement of pyruvate

The HPLC measurement of pyruvate followed a previously reported method with modifications^14^. Samples and pyruvate standards were derivatized by combining 1:1 by volume with O-phenylenediamine (OPD) dissolved at 25 mM in 2 M HCl, and incubated at 80 °C for 30 min. Derivatized pyruvate was quantified by HPLC with fluorescence detection using an Agilent 1260 Infinity II system with binary pump (G7112B) and fluorescence detector (G7121A). Samples were separated using an InfinityLab Poroshell 120 HPH C18 column (2.1 ×100 mm, 2.7 µm) with a guard column. Mobile phase A was 0.1% formic acid in water, and mobile phase B was 0.1% formic acid in acetonitrile. The injection volume was 5 µL. The mobile phase flow rate was 420 µL/min. The autosampler sample compartment was maintained at 4 °C, and the column oven was set at 40 °C. The mobile phase gradient (%B) was 0 min, 5%; 11 min, 60%; 11.1 min, 5%; 14.1 min, 5%. The fluorescence λ_ex_ was 350 nm and λ_em_ 410 nm with 100 LU attenuation. Peak areas were integrated using OpenLab CDS ChemStation (Agilent) and sample pyruvate concentrations calculated from a standard curve.

### LC-MS measurement of pyruvate and acetate concentrations

LC-MS analysis was performed on a Vanquish ultra-high performance liquid chromatography system coupled to a Q Exactive mass spectrometer (Thermo) that was equipped with an Ion Max source and HESI II probe adapting previously described methods^24,25^. External mass calibration was performed every seven days. Metabolites were separated using a ZIC-pHILIC stationary phase column (2.1 ×150 mm, 5 µm; Merck) with a guard column. Mobile phase A was 20 mM ammonium carbonate and 0.1% ammonium hydroxide. Mobile phase B was acetonitrile. The injection volume was 1 µL, the mobile phase flow rate was 100 µL/min, the column compartment temperature was set at 25 °C, and the autosampler compartment was set at 4 °C. The mobile phase gradient (%B) was 0 min, 80%; 10 min, 50%; 10.5 min, 8%; 14 min, 8%; 14.5 min, 80%; 25 min, 80%. The column effluent was introduced to the mass spectrometer with the following ionization source settings: sheath gas 40, auxiliary gas 15, sweep gas 1, spray voltage - 3.0 kV, capillary temperature 275 °C, S-lens RF level 40, probe temperature 350 °C. The mass spectrometer was operated in targeted selective ion monitoring mode for pyruvate (m/*z* 87.0088) and acetate (m/*z* 59.0135) with 2 m/*z* isolation window. The resolution was set to 140,000, and the AGC target was 3 × 10^6^ ions. Data were acquired and analyzed using TraceFinder software (Thermo). Metabolite concentrations were calculated from the corresponding standard curve.

### Amplex red assay

An Amplex® Red Hydrogen Peroxide/Peroxidase Assay Kit was used for the H_2_O_2_ concentration measurement, following the manufacturer’s instruction with modifications. Hydrogen peroxide standards and samples (10–50 µL) were loaded in triplicate into a 96 well black polystyrene microplate with a clear bottom (Corning). A working solution was immediately added for a final volume of 100 µL after each triplicate loading. The final concentration of horseradish peroxidase in the mixture was 0.1 U/mL, and Amplex Red was 0.05 mM. After 5–10 min incubation at room temperature in the dark, the plate was read with a microplate reader, SpectraMax MiniMax 300 Imaging Cytometer, equipped with SoftMax Pro 7 software (Molecular Devices). Fluorescence detection was set at λ_ex_ 540 nm and λ_em_ 580 nm and optical absorbance detection at 560 nm. Sample concentrations were calculated according to standard curves included in each experiment.

### Statistics

Data were obtained from 3–6 replicate experiments, each of which was performed in duplicate or triplicate. Data are presented as mean ± standard deviation. Curve fitting was performed with nonlinear least-squares regression, and R^2^ of the fitting is presented when necessary in figure plots.
